# Developmental dyslexia genes are selectively targeted by diverse environmental pollutants

**DOI:** 10.1186/s12888-024-05952-4

**Published:** 2024-07-17

**Authors:** Yangyang Yang, Tingting Zheng, Qidi Tang, Bing Xiang, Mei Yang, Jing Zeng, Feng Zhou, Xinyan Xie

**Affiliations:** 1 Research Center for Health Promotion in Women, Youth and Children, Hubei Province Key Laboratory of Occupational Hazard Identification and Control, School of Public Health, Wuhan University of Science and Technology, West Huangjiahu Road, Hongshan District, Wuhan, 430065 China; 2 Department of Cardiology, Renmin Hospital of Wuhan University, Wuhan, 430060 China

**Keywords:** Developmental dyslexia, Neurodevelopmental disorder, Environmental compounds, Chemical bias, Dyslexia genes

## Abstract

**Background:**

Developmental dyslexia, a complex neurodevelopmental disorder, not only affects children’s academic performance but is also associated with increased healthcare costs, lower employment rates, and reduced productivity. The pathogenesis of dyslexia remains unclear and it is generally considered to be caused by the overlap of genetic and environmental factors. Systematically exploring the close relationship between exposure to environmental compounds and susceptibility genes in the development of dyslexia is currently lacking but high necessary.

**Methods:**

In this study, we systematically compiled 131 publicly reported susceptibility genes for dyslexia sourced from DisGeNET, OMIM, and GeneCards databases. Comparative Toxicogenomics Database database was used to explore the overlap between susceptibility genes and 95 environmental compounds, including metals, persistent organic pollutants, polycyclic aromatic hydrocarbons, and pesticides. Chemical bias towards the dyslexia risk genes was taken into account in the observation/expectation ratios > 1 and the corresponding *P* value obtained by hypergeometric probability test.

**Results:**

Our study found that the number of dyslexia risk genes targeted by each chemical varied from 1 to 109. A total of 35 chemicals were involved in chemical reactions with dyslexia-associated genes, with significant enrichment values (observed/expected dyslexia risk genes) ranging from 1.147 (Atrazine) to 66.901 (Dibenzo(a, h)pyrene).

**Conclusion:**

The results indicated that dyslexia-associated genes were implicated in certain chemical reactions. However, these findings are exploratory, and further research involving animal or cellular experiments is needed.

**Supplementary Information:**

The online version contains supplementary material available at 10.1186/s12888-024-05952-4.

## Introduction

Developmental dyslexia is a specific learning disability that is characterized by difficulties with accurate and/or fluent word recognition and by poor spelling and decoding abilities. Due to differences in language and writing systems, the prevalence of reading disorders varies among different countries, generally ranging from 3.45 to 17.5% [1, 2]. Dyslexia not only affects children’s academic performance, but is also associated with increased healthcare costs, lower employment rates, and reduced productivity. As a complex neurodevelopmental disorder, the pathogenesis of dyslexia is still unclear and is generally considered to be caused by the interaction of genetic and environmental factors [3].

Researchers have focused on the neurodevelopmental impacts of chemical exposures in children, as children are more sensitive to exposure to chemicals due to their physiological characteristics, such as faster metabolism, higher relative exposure per unit body weight than adults, and incomplete immune function [4]. Agency for Toxic Substances and Disease Registry (ATSDR) has reported peer-reviewed toxicological profiles for 185 classes of chemicals [5], of which organophosphate (OP) pesticides, air pollutants, polybrominated diphenyl ether flame retardants, lead, mercury, and polychlorinated biphenyls were common chemicals that could increase the risk of neurodevelopmental disorders in children [6]. Increasing numbers of epidemiologic, clinical, and animal studies have demonstrated that early chemical exposures were associated with several adverse outcomes, such as autism spectrum disorder (ASD) [7, 8], attention-deficit hyperactivity disorder (ADHD) [9, 10], and intellectual disability [11]. Neurotoxic chemicals could impact brain development through multiple mechanisms, including oxidative stress effects, neurotransmitter effects, neuroendocrine effects, immune effects, and behavioral phenotype [12]. For example, exposure to poly chlorinated biphenyls (PCBs) and dibenzo-p-dioxins could change thyroid function and reduce the level of thyroid hormone, an important regulator of brain development, resulting in abnormal brain development [13]. Arsenic exposure may change the central pathways involved in regulating learning and memory in hippocampus, such as Ras-MAPK/ERK pathway, which may be the basis of arsenic-induced behavioral defects, thus leading to abnormal brain development [14].

An increasing number of studies are beginning to explore the association between exposure to chemicals and dyslexia. So far, eight epidemiological studies have suggested a significant association between exposure to metals [15–18], organophosphate (OP) insecticides [19], propylene oxide [20], and sodium iodide symporter (NIS) inhibitors [21] with the risk of dyslexia (summarized in Table S1). However, no similar results have been found for air pollutants [22]. The mechanism by which chemical compound exposure led to dyslexia was still unclear, but it was possible that these compounds could overlap with genes, thereby affecting the normal development and function of the nervous system and cognitive function. Zinc, a trace element that plays an important role in neurological disorders, was associated with dyslexia risk mediated by the *GRIN2B* gene polymorphism rs1805502 [23]. Similarly, mutation in *SLC6A3* rs27072 could impact the association between urine manganese levels and the risk of dyslexia [18]. Systematically exploring the close relationship between exposure to environmental compounds and susceptibility genes in the development of dyslexia is currently lacking but high necessary, which may contribute a lot to the etiological research of dyslexia.

Thus, in our current study, we systematically compiled publicly reported susceptibility genes for dyslexia and used the Comparative Toxicogenomics Database (CTD) [24] database to explore the overlap between susceptibility genes and environmental compounds, including metals, persistent organic pollutants (POPs), polycyclic aromatic hydrocarbons (PAHs), and pesticides. We aimed to identify chemical compounds involved in dyslexia susceptibility genes.

## Methods

### Dyslexia risk genes

The dyslexia risk genes were derived from DisGeNET [25] (*n* = 110), OMIM (*n* = 7), and GeneCards databases (*n* = 72). DisGeNET integrates data from expert curated repositories, GWAS catalogues, animal models, and the scientific literature. The current version of DisGeNET (v7.0) contains 1,134,942 gene-disease associations, between 21,671 genes and 30,170 diseases, disorders, characteristics, and clinical or abnormal human phenotypes. OMIM is a comprehensive and authoritative outline of human genes and genetic phenotypes, which is provided free of charge and updated daily. After removing duplicates, a total of 131 dyslexia risk genes were included in this study (0.69%) from a human genome of 19,000 protein-coding genes [26]. The detail information of genes was shown in Table S2.

### Chemical-gene overlap

The gene symbols were uploaded to the CTD database [24] (http://ctdbase.org/). There are 14,489 unique chemicals in CTD, with 2,674,978 curated chemical-gene overlap (2023 data). The genes influenced by each chemical in CTD are determined using curated data from diverse studies, including gene expression studies, protein-protein interaction assays, gene knockout experiments, and computational analyses. After downloading the results, we focused on three types of data. One is the number of genes involved in chemical reactions, the other is the number of dyslexia-related genes involved in chemical reactions, and the last is how many chemical reactions each dyslexia-related gene may participate in. We obtained the chemical list from United States Environmental Protection Agency (https://www.epa.gov/) and National Pesticide Information Center (http://npic.orst.edu/). We removed chemicals that lacked relevant information or evidence of gene involvement in chemical reactions. A total of 95 chemicals in 7 classes were included in this study, including metals (*n* = 19), POPs (*n* = 12), PAHs (*n* = 23), herbicides (*n* = 9), insecticides (*n* = 10), fungicides (*n* = 15), and rodenticides (*n* = 7) (Table 1). For the joint effect between the compounds we were interested in and susceptibility genes for dyslexia, the evidence from studies in CTD showed that there were 51,281 cases (48.9%) involving Homo sapiens, 25,317 cases (24.1%) involving Mus musculus, and Rattus norvegicus data ranked third, totaling 9525 cases (9.0%).

**Table 1:** Chemicals involved in the current study

Classes	Chemicals
Metals	Aluminum, vanadium, chromium, manganese, iron, cobalt, nickel, copper, zinc, arsenic, selenium, rubidium, strontium, cadmium, cesium, barium, mercury, thallium, and lead
Persistent organic pollutants (POPs)	Aldrin, chlordane, dichlorodiphenyl trichloroethane, dieldrin, endrin, heptachlor, hexachlorobenzene, mirex, toxaphene, polychlorinated biphenyls, polychlorinated dibenzo-p-dioxins, and polychlorinated dibenzofurans
Polycyclic aromatic hydrocarbons (PAHs)	Benzo(a)anthracene, benzo(a)phenanthrene, benzo(b)fluoranthene, benzo(j)fluoranthene, benzo(k)fluoranthene, benzo(j, k)fluorene, dibenzacridine, dibenzo(a, h)anthracene, dibenzo(a, e)pyrene, dibenzo(a, h)pyrene, dibenzo(a, l)pyrene, 7 h-dibenzo(c, g)carbazole, 7,12-dimethylbenz(a)anthracene, indeno(1,2,3-cd)pyrene,3-methylcholanthrene, 5-methylchrysene, 1-nitropyrene, acenaphthene, anthracene, benzo(g, h,i)perylene, fluorene, phenanthrene, and pyrene
Herbicides	Glyphosate, imazethapyr, atrazine, cyanazine, 2,4-d, dicamba, trifluralin, pendimethalin, and metolachlor
Insecticides	Cyfluthrin, tefluthrin, aldicarb, lambda-cyhalothrin, permethrin, terbufos, chlorpyrifos, methyl parathion, dimethoate, and carbofuran
Fungicides	Hexachlorobenzene, pentachloronitrobenzene, chlorothalonil, metam, ferbam, sodium, ziram, mancozeb, maneb, zineb benomyl, cycloheximide, triadimefon, metalaxyl, and thiabendazole
Rodenticides	Warfarin, diphacinone, bromadiolone, difethialone, brodifacoum, cholecalciferol, and strychnine

### Gene enrichment analysis

If N genes were involved in a chemical reaction, one would expect an equal proportion of dyslexia risk genes (0.69%) to be contained within gene set (Expected = N × (131/19,000). Chemical bias towards the dyslexia risk genes was taken into account in the observation/expectation ratios > 1 and the corresponding *P* value obtained by hypergeometric probability test. *P* value was corrected for false discovery within each category group of chemicals, with a final cut-off at *P* < 0.05. Analyses were conducted using R software version 4.1.0 (R Foundation for Statistical Computing).

### Sensitivity analysis

Considering that dyslexia involves brain-mediated effects, we restricted the analysis to genes expressed in the brain. We obtained gene expression data from the Genotype-Tissue Expression Project (GTEx) and discovered that all 131 dyslexia-related genes were expressed in various regions of the brain, except for the *STATH* gene. The *STATH* gene, located in the 4q13.3 region and consisting of 5 exons, encodes a protein called statherin, which possesses antibacterial properties and is expressed in saliva, the upper respiratory tract, and nasal secretions [27].

## Results

The number of dyslexia risk genes targeted by each chemical varied from 1 to 109 (Polychlorinated dibenzo-p-dioxins, belonging to PAHs). Nineteen chemicals did not overlap with any dyslexia gene. The number of chemicals involved in dyslexia-related genes ranged from 1 to 532 (*PARP1*) (Table S1). There were 35 chemicals with significant enrichment values (observed/expected dyslexia risk genes) involved in chemical reactions with dyslexia-associated genes, ranging from 1.147 (Atrazine) to 66.901 (Dibenzo(a, h)pyrene).

### Metals

Of 19 metals, 10 showed significant enrichment values in relation to the dyslexia risk genes. The enrichment values, which reflected the chemical bias towards the dyslexia risk gene were 1.479, 2.877, 1.325, 1.253, 1.499, 1.237, 1.623, 4.956, 2.739, and 1.718 respectively for chromium (number of dyslexia genes/ all targeted genes: 26/2352, 1.479 fold, *P*_FDR_ =0.017), manganese (12/558, 2.877 fold, *P*_FDR_ =0.002), cobalt (48/4849, 1.325 fold, *P*_FDR_ =0.008), copper (58/6193,1.253 fold, *P*_FDR_ =0.008), zinc (30/2678, 1.499 fold, *P*_FDR_ =0.008), arsenic (46/4977, 1.237 fold, *P*_FDR_ =0.022), selenium (24/1978, 1.623 fold, *P*_FDR_ =0.008), barium (3/81, 4.956 fold, *P*_FDR_ =0.027), mercury (13/635, 2.739 fold, *P*_FDR_ =0.002) and lead (42/3271,1.718 fold, *P*_FDR_ =2.76E-04). We found that rubidium, strontium, and cesium did not target any dyslexia genes (Table 2).

**Table 2:** The number of dyslexia risk genes affected by metals

Metal	Numbers of all genes affected	Numbers of dyslexia risk genes affected	Expected^a^	Ratio^b^	*P* value^c^	*P*_FDR_ value^d^
Aluminum	1937	16	14.477	1.105	0.249	0.266
Vanadium	182	1	1.360	0.735	0.716	0.716
Chromium	2352	26	17.578	**1.479**	0.008	**0.017**
Manganese	558	12	4.170	**2.877**	4.01E-04	**0.002**
Iron	458	7	3.423	2.045	0.037	0.054
Cobalt	4849	48	36.240	**1.325**	0.003	**0.008**
Nickel	7653	62	57.196	1.084	0.051	0.063
Copper	6193	58	46.285	**1.253**	0.003	**0.008**
Zinc	2678	30	20.015	**1.499**	0.004	**0.008**
Arsenic	4977	46	37.197	**1.237**	0.012	**0.022**
Selenium	1978	24	14.783	**1.623**	0.004	**0.008**
Rubidium	15	0	0.112	-	-	-
Strontium	21	0	0.157	-	-	-
Cadmium	5266	45	39.356	1.143	0.050	0.063
Cesium	29	0	0.217	-	-	-
Barium	81	3	0.605	**4.956**	0.017	**0.027**
Mercury	635	13	4.746	**2.739**	3.70E-04	**0.002**
Thallium	62	2	0.463	4.316	0.064	0.074
Lead	3271	42	24.446	**1.718**	1.73E-05	**2.76E-04**

^a^ If a chemical affects N genes, one would expect an equal proportion of dyslexia risk gene (0.69%) to be contained within this gene set (Expected = N × (131/19,000))

^b^ Representing the enrichment value which was obtained by dividing the observed value by the expected value

^c^ The corresponding *P* value of observed/expected ratios derived from the hypergeometric probability test

^d^*P* value was corrected for false discovery

*P*_FDR_ value was bold if *P*_FDR_ was less than 0.05

### Persistent organic pollutants

Of 12 POPs, 5 showed significant enrichment values in relation to the dyslexia risk genes. Chlordane (number of dyslexia genes/ all targeted genes: 5/215, 3.112 fold, *P*_FDR_ = 0.037), dichlorodiphenyl trichloroethanen (21/1160, 2.422 fold, *P*_FDR_ = 2.02E-04), dieldrin (25/1494, 2.239 fold, *P*_FDR_ = 2.02E-04), heptachlor (7/229, 4.090 fold, *P*_FDR_ = 0.004), and toxaphene (4/80, 6.690 fold, *P*_FDR_ = 0.006) showed significant bias towards dyslexia risk genes (Table 3).

**Table 3:** The number of dyslexia risk genes affected by persistent organic pollutants (POPs)

Persistent organic pollutants (POPs)	Numbers of all genes affected	Numbers of dyslexia risk genes affected	Expected^a^	Ratio^b^	*P* value^c^	*P*_FDR_ value^d^
Aldrin	225	3	1.682	1.784	0.198	0.216
Chlordane	215	5	1.607	**3.112**	0.015	**0.037**
Dichlorodiphenyl trichloroethane	1160	21	8.669	**2.422**	3.36E-05	**2.02E-04**
Dieldrin	1494	25	11.166	**2.239**	2.10E-05	**2.02E-04**
Endrin	86	2	0.643	3.112	0.114	0.137
Heptachlor	229	7	1.711	**4.090**	0.001	**0.004**
Hexachlorobenzene	224	4	1.674	2.389	0.066	0.099
Mirex	53	2	0.396	5.049	0.048	0.097
Toxaphene	80	4	0.598	**6.690**	0.002	**0.006**
Polychlorinated biphenyls	9137	72	68.287	1.054	0.057	0.098
Polychlorinated dibenzo-p-dioxins	17,068	109	127.561	0.854	0.988	0.988
Polychlorinated dibenzofurans	75	2	0.561	3.568	0.090	0.121

^a^ If a chemical affects N genes, one would expect an equal proportion of dyslexia risk gene (0.69%) to be contained within this gene set (Expected = N × (131/19,000))

^b^ Representing the enrichment value which was obtained by dividing the observed value by the expected value

^c^ The corresponding *P* value of observed/expected ratios derived from the hypergeometric probability test

^d^*P* value was corrected for false discovery

*P*_FDR_ value was bold if *P*_FDR_ was less than 0.05

### Polycyclic aromatic hydrocarbons

Of 23 PAHs, 7 medicals did not target any dyslexia genes. The enrichment value of dibenzo(a, h)pyrene had reached 69, but it should be viewed with caution because it could only target two protein-coding genes in the genome, one of which was a dyslexia risk gene. Anthracene (number of dyslexia genes/ all targeted genes: 3/21, 19.115 fold, *P*_FDR_ = 0.002), and phenanthrene (7/154, 6.082 fold, *P*_FDR_ = 0.001) showed significant bias towards dyslexia risk genes (Table 4).

**Table 4:** The number of dyslexia risk genes affected by polycyclic aromatic hydrocarbons (PAHs)

Polycyclic Aromatic Hydrocarbons	Numbers of all genes affected	Numbers of dyslexia risk genes affected	Expected^a^	Ratio^b^	*P* value^c^	*P*_FDR_ value^d^
Benzo(a)anthracene	1413	12	10.560	1.136	0.259	0.411
Benzo(a)phenanthrene	954	8	7.130	1.122	0.328	0.438
Benzo(b)fluoranthene	1684	12	12.586	0.953	0.487	0.599
Benzo(j)fluoranthene	5	0	0.037	-	-	-
Benzo(k)fluoranthene	753	7	5.628	1.244	0.256	0.411
Benzo(j, k)fluorene	956	12	7.145	1.680	0.030	0.080
Dibenzacridine	4	0	0.030	-	-	-
Dibenzo(a, h)anthracene	916	6	6.846	0.876	0.603	0.689
Dibenzo(a, e)pyrene	3	0	0.022	-	-	-
Dibenzo(a, h)pyrene	2	1	0.015	**66.901**	0.007	**0.037**
Dibenzo(a, l)pyrene	394	4	2.945	1.358	0.282	0.411
7 H-Dibenzo(c, g)carbazole	2	0	0.015	-	-	-
7,12-Dimethylbenz(a)anthracene	1304	16	9.746	1.642	0.016	0.063
Indeno(1,2,3-cd)pyrene	255	5	1.906	2.624	0.030	0.080
3-Methylcholanthrene	1305	8	9.753	0.820	0.678	0.724
5-Methylchrysene	4	0	0.030	-	-	-
1-Nitropyrene	263	1	1.966	0.509	0.839	0.839
Acenaphthene	9	0	0.067	-	-	-
Anthracene	21	3	0.157	**19.115**	2.42E-04	**0.002**
Benzo(g, h,i)perylene	290	5	2.167	2.307	0.048	0.107
Fluorene	16	0	0.120	-	-	-
Phenanthrene	154	7	1.151	**6.082**	7.21E-05	**0.001**
Pyrene	300	5	2.242	2.230	0.054	0.107

^a^ If a chemical affects N genes, one would expect an equal proportion of dyslexia risk gene (0.69%) to be contained within this gene set (Expected = N × (131/19,000))

^b^ Representing the enrichment value which was obtained by dividing the observed value by the expected value

^c^ The corresponding *P* value of observed/expected ratios derived from the hypergeometric probability test

^d^*P* value was corrected for false discovery

*P*_FDR_ value was bold if *P*_FDR_ was less than 0.05

### Pesticides

We focused on 4 types of pesticides (41 chemicals), including herbicides, insecticides, fungicides, and rodenticides. Nine chemicals did not target any dyslexia genes. Of 9 herbicides, 6 showed significant enrichment values in relation to the dyslexia risk genes. Dyslexia-related genes may participate in the chemical reactions of atrazine, chlorpyrifos, cholecalciferol, and permethrin, with 72, 41, 32, and 27 genes participating in the chemical reactions respectively. The enrichment values ranged from 1.147 to 44.601. Of 10 insecticides, cyfluthrin (number of dyslexia genes/ all targeted genes:3/88, 4.561 fold, *P*_FDR_ =0.038), lambda-cyhalothrin (8/210, 5.097 fold, *P*_FDR_ =2.41E-04), permethrin (27/1318, 2.741 fold, *P*_FDR_ =1.58E-04), chlorpyrifos (41/3349, 1.638 fold, *P*_FDR_ =5.41E-04) and dimethoate (3/62,6.474 fold, *P*_FDR_ =0.018) were more likely to act on dyslexia risk genes. Of 15 fungicides, maneb (11/459, 3.207 fold, *P*_FDR_ = 0.003), benomyl (3/62, 6.474 fold, *P*_FDR_ = 0.029) and cycloheximide (11/501, 2.938 fold, *P*_FDR_ = 0.003) showed significant bias towards dyslexia risk genes. Of 7 rodenticides, brodifacoum (12/660, 2.433 fold, *P*_FDR_ =0.005) and cholecalciferol (32/2830, 1.513 fold, *P*_FDR_ =0.005) were rodenticides that were more likely to participate in dyslexia-related genes (Table 5).

**Table 5:** The number of dyslexia risk genes affected by pesticides

Pesticides	Numbers of all genes affected	Numbers of dyslexia risk genes affected	Expected^a^	Ratio^b^	*P* value^c^	*P*_FDR_ value^d^
Herbicides						
Glyphosate	1831	25	13.684	**1.827**	0.001	**0.002**
Imazethapyr	1	0	0.007	-	-	-
Atrazine	8397	72	62.757	**1.147**	0.007	**0.010**
Cyanazine	12	4	0.090	**44.601**	1.48E-07	**1.18E-06**
2,4-D	153	3	1.143	2.624	0.085	0.086
Dicamba	14	1	0.105	9.557	0.086	0.086
Trifluralin	18	2	0.135	**14.867**	0.005	**0.010**
Pendimethalin	21	2	0.157	**12.743**	0.007	**0.010**
Metolachlor	72	4	0.538	**7.433**	0.001	**0.003**
**Insecticides**						
Cyfluthrin	88	3	0.658	**4.561**	0.021	**0.038**
Tefluthrin	10	0	0.075	-	-	-
Aldicarb	810	1	6.054	0.165	0.997	0.997
Lambda-cyhalothrin	210	8	1.569	**5.097**	8.04E-05	**2.41E-04**
Permethrin	1318	27	9.850	**2.741**	1.76E-07	**1.58E-06**
Terbufos	733	1	5.478	0.183	0.994	0.997
Chlorpyrifos	3349	41	25.029	**1.638**	7.14E-05	**5.41E-04**
Methyl parathion	101	5	0.755	6.624	0.800	0.997
Dimethoate	62	3	0.463	**6.474**	0.008	**0.018**
Carbofuran	120	2	0.897	2.230	0.196	0.293
**Fungicides**						
Hexachlorobenzene	224	4	1.674	2.389	0.066	0.081
Pentachloronitrobenzene	8	0	0.060	-	-	-
Chlorothalonil	93	1	0.695	1.439	0.472	0.519
Metam	105	0	0.785	-	-	-
Ferbam	3	1	0.022	**44.601**	0.014	**0.038**
Sodium	89	0	0.665	-	-	-
Ziram	157	1	1.173	0.852	0.662	0.662
Mancozeb	121	3	0.904	3.317	0.048	0.072
Maneb	459	11	3.430	**3.207**	0.000	**0.003**
Zineb	125	3	0.934	3.211	0.052	0.072
Benomyl	62	3	0.463	**6.474**	0.008	**0.029**
Cycloheximide	501	11	3.744	**2.938**	0.001	**0.003**
Triadimefon	492	7	3.677	1.904	0.051	0.072
Metalaxyl	3	0	0.022	-	-	-
Thiabendazole	337	6	2.519	2.382	0.027	0.060
**Rodenticides**						
Warfarin	748	5	5.590	0.894	0.585	0.585
Diphacinone	1	0	0.007	-	-	-
Bromadiolone	14	1	0.105	9.557	0.086	0.115
Difethialone	4	0	0.030	-	-	-
Brodifacoum	660	12	4.933	**2.433**	0.002	**0.005**
Cholecalciferol	2830	32	21.151	**1.513**	0.002	**0.005**
Strychnine	37	0	0.277	-	-	-

^a^ If a chemical affects N genes, one would expect an equal proportion of dyslexia risk gene (0.69%) to be contained within this gene set (Expected = N × (131/19,000))

^b^ Representing the enrichment value which was obtained by dividing the observed value by the expected value

^c^ The corresponding *P* value of observed/expected ratios derived from the hypergeometric probability test

^d^*P* value was corrected for false discovery

*P*_FDR_ value was bold if *P*_FDR_ was less than 0.05

### Sensitivity analysis

After excluding *STATH* from the list of dyslexia risk genes, we meticulously analyzed the data. Remarkably, the results remained consistent, demonstrating that 35 chemicals exhibited a significant bias towards dyslexia risk genes (detailed data not shown in the Tables).

## Discussion

The present study aimed to evaluate the overlap effect between 95 chemicals and 131 dyslexia risk genes. Findings from this study added some evidence that chemicals may act on dyslexia-related genes and thus play a role in the occurrence of the condition. We found that the dyslexia-related genes have significant chemical reactions with some chemicals belonging to metals, POPs, PAHs, and pesticides. Our study provides only exploratory insights into the mechanism by which chemicals may contribute to the development of dyslexia, and further research involving animal or cellular experiments is needed.

Research on metals and their potential role in dyslexia has been growing in recent years. Previous population-based studies have identified several metals, including lead, copper, zinc, selenium, and argentum as potential contributors to the risk of dyslexia [16, 17]. Heavy metals are known to be neurotoxic and could have negative effects on neurodevelopment [28]. Studies have shown that prenatal and early childhood exposure to heavy metals could impair cognitive development, language skills, attention, and behavior [29]. Children exposed to lead, for example, showed neurobehavioral deficits in several areas, such as intelligence, attention, and executive function [30]. Arsenic exposure has been linked to reduced cognitive function and increased risk of ADHD [31]. While other metals, such as selenium and zinc, have been implicated in exerting neuroprotective effects through their involvement in defense mechanisms against oxidative stress [17]. Metals could affect normal brain development by interfering with the formation and function of neurons, glial cells, and synapses [4]. The overlapping effect between environment and gene has been established to be a strong determinant of neurodevelopmental disorders. The synergistic impact of excessive manganese exposure and a genetic modification in the *GSTP1* gene may potentiate the incidence of mitochondrial dysfunction and oxidative stress, both of which have been documented as underlying mechanisms contributing to ASD [28]. Excessive Cu exposure could lead to the accumulation of Cu in the brain tissue of mice. Cu damages synaptic plasticity through CREB/BDNF pathway, stimulates copper proliferation, promotes cell death, and causes learning and memory disorders in mice [32]. This current study adds to this body of existing research by identifying additional metals, including chromium, cobalt, copper, zinc, arsenic, selenium, and barium. Susceptible dyslexia genes may be involved in the reaction of these metals. The findings suggest that these metals may contribute to the occurrence of dyslexia by altering the expression of relevant genes.

Our study revealed a potential association between a group of 5 POPs and dyslexia-associated genes. The 109 dyslexia-associated genes may be involved in the chemical reaction of polychlorinated dibenzo-p-dioxins, although the *P* value obtained by hypergeometric probability test is not significant. PCDDs are unintentionally by-products generated during the manufacture and combustion process involving chlorine or chlorine-derived chemicals [33]. We observed significant biases towards dyslexia risk genes in the case of chlordane, dichlorodiphenyl trichloroethane, dieldrin, heptachlor, and toxaphene. Epidemiological research has indicated that higher maternal levels of POP exposures during pregnancy were associated with increased autism-related behaviors, poorer cognitive function, and reduced adaptive function [34]. POPs could affect the symptom domain of ASD by acting on SNP site of *ESR1* gene [35, 36]. Synapse appears to be a common susceptible target of POPs, altering dendrite and dendritic spine morphology, as well as synaptic function during sensitive developmental periods, leading to cognitive and behavioral dysfunction [37]. Research has provided evidence indicating that prenatal exposure to p, p’-DDT and DDE may have detrimental effects on the neurological development of children. High levels of DDE detected in maternal serum or umbilical cord blood serum during pregnancy were believed to be associated with reduced cognitive function, including language skills, spatial orientation, and memory. Additionally, Dieldrin has been found to induce oxidative stress and trigger mitochondrial-mediated apoptosis [38]. Heptachlor, functioning as a direct neurotoxic agent, could impact dopaminergic neurons, resulting in damage to both dopaminergic neurons and brain nerve function. Its pathogenesis may be associated with the overlap of the dyslexia gene *DRD2*, although further investigation is required to confirm this relationship [39]. Our findings provide valuable insights into the potential association between POPs and dyslexia, suggesting a role for these substances in dyslexia pathogenesis. Further investigations are warranted to elucidate the underlying mechanisms and overlaps between these POPs and dyslexia. Understanding how these substances influence the expression and function of dyslexia-associated genes will contribute to a comprehensive understanding of dyslexia pathogenesis and provide scientific evidence for preventive and therapeutic interventions. Additionally, it is crucial to consider the interplay of other potential factors, such as genetic and environmental factors, to comprehensively assess the risk of dyslexia occurrence.

Our study showed that 3 of the PAH compounds exhibited a significant bias against dyslexia genes. Previous studies have shown that the widespread use of synthetic chemicals (including fuels, refrigerants, lubricants, and solvents, among others) has significantly increased in recent years, potentially contributing to the rise in neurodevelopmental disorders such as ASD and ADHD [40]. PAHs may impact cognitive function by reducing plasma levels of brain-derived neurotrophic factor (BDNF) [41]. BDNF is one of the most plentiful neurotrophic factors in the central nervous system [42, 43]. Synthesized and secreted by neurons in the brain, it plays a vital role in neuronal survival, differentiation, development, hippocampal neurogenesis, synaptic plasticity, and cognitive function regulation [41]. PAHs might decrease the expression of BDNF mRNA by potentially influencing the negative regulation of the *BDNF-AS* gene on BDNF. These effects may lead to abnormalities in synaptic function associated with cognitive functions in the brain, resulting in impaired transmission of nerve impulses and subsequent cognitive impairment, and may also increase the risk of ADHD [44]. These alterations may be observed as reductions in the surface area of children’s white matter, as well as decreases in their head circumference, birth weight, and birth length [45]. However, the specific mechanism underlying these effects remains unclear, highlighting the need for further research in this area.

We also identified potential biases against dyslexia-related genes in 6 out of the 9 herbicides studied. Glyphosate, the active ingredient in glyphosate-based herbicides (GBHs), such as Roundup™, is widely used worldwide as an herbicide. Experimental evidence suggests that exposure to glyphosate can impact synaptic transmission and induce morphological and biochemical changes, which may contribute to cognitive impairment [46]. Studies have demonstrated that the molecular mechanism of glyphosate-induced neurotoxicity may be linked to the imbalance of miRNA expression. The miRNA regulates the target mRNA, thus affecting the activities of various cells and tissues. Glyphosate, the active ingredient in glyphosate-based herbicides (GBHs), such as Roundup™, is widely used worldwide as an herbicide. Experimental evidence suggests that exposure to glyphosate can impact synaptic transmission and induce morphological and biochemical changes, which may contribute to cognitive impairment [46]. The *SCL6A3* gene may react with glyphosate exposed to zebrafish, and the expression of *SCL6A3* is down-regulated, and the oxidative stress in zebrafish is increased, thus showing obvious damage to exploration and social behavior [47]. Studies have demonstrated that the molecular mechanism of glyphosate-induced neurotoxicity may be linked to the imbalance of miRNA expression. The miRNA regulates the target mRNA, thus affecting the activities of various cells and tissues. Forkhead box G1 (*FOXG1*) is one of the target genes implicated in these processes through miR-34b-5β [48]. *FOXG1*, along with *FOXP2*, a gene associated with dyslexia, belongs to the FOX gene family, and its encoded transcription factor is specifically expressed in fetal and adult brain tissues. Mutation of *FOXP2* gene could also affect general language ability, which lead to speech and facial dyskinesia [49]. Moreover, an experiment demonstrated that exposure of both young and old mice to Atrazine resulted in the production of pro-inflammatory cytokines in the prefrontal cortex and hippocampus, accompanied by a significant decrease in interleukin-10 release. Additionally, exposure to varying levels of Atrazine was found to potentially modify the neurodevelopment of zebrafish by altering protein abundance [50]. The exposure of children to pesticides affects their brain development, leading to impaired cognitive functions. This pathogenesis may be associated with dyslexia-related gene expression or changes in protein abundance. These findings provide valuable insights for further research on developmental dyslexia.

Significant biases towards dyslexia-related genes in 5 out of the 10 pesticide species examined were identified. Current research provides evidence that exposure to various pesticides (including organophosphorus and parathyroid pesticides) during pregnancy could disrupt gene pathways at the placental and brain levels, potentially impacting fetal brain development and increase the risk of mental illness such as ASD and intellectual disability (ID) [51, 52]. Epidemiological studies have shown that early childhood exposure to pesticides was associated with cognitive decline and behavioral problems [53]. Pesticides exposure could increase the activity of *PARP1* expression, promote the death of nerve cells, affect the signal transduction of neurons, and lead to synaptic and behavioral changes related to neurodevelopmental disorders (NDDs) [52]. Organophosphorus pesticides, a commonly used pesticide group, could inhibit the activity of cholinesterase, thereby disrupting nerve signal transmission and causing dysfunction in the nervous system. Children affected by pesticide poisoning may experience difficulties in concentration, verbal IQ, and cognitive impairment related to graphics. Dimethoate, like other organophosphorus pesticides, inhibits acetylcholinesterase, leading to an accumulation of acetylcholine at synapses and neuromuscular junctions, resulting in over-activation of nicotine and noxious alkali receptors and disruption of neurotransmitters. Lambda-cyhalothrin induces a significant increase in malondialdehyde levels, a metabolite associated with oxidative stress, in kidney and brain tissues, leading to tissue damage [54]. Among the 15 fungicides studied, 4 displayed significant biases against dyslexia genes. The high manganese concentration in Maneb, which could be neurotoxic to exposed individuals, may be related to altered patterns of brain activation in the prefrontal cortex, an area involved in working memory [55]. Two out of the 7 rodenticides examined exhibited significant biases against dyslexia risk genes. Rodenticide poisoning could result in symmetrical lesions in the white matter and corpus callosum of the brain, leading to cognitive impairment [56]. Our findings suggest that different pesticides have distinct mechanisms of action in the pathogenesis of brain nerves, which could damage the brain by altering gene expression, inducing oxidative stress, and impairing mitochondrial function. These findings provide some insights into the potential relationship between pesticides and dyslexia. Pesticides may contribute to the pathogenesis of dyslexia, but further research is needed to clarify this association.

Our findings suggested a potential correlation between exposure to environmental compounds and susceptibility genes in the onset of dyslexia. Prior research has hinted at the potential involvement of neuro/immune biological mechanisms in mediating these effects [57]. Through examining dyslexia susceptibility genes that were notably enriched with chemical-related genes, we delved into their possible roles within neuro/immune biological processes. Our results revealed that 14 genes, constituting 10.7% of the total, significantly influenced various aspects of these pathways. For instance, *BDNF* may be associated with the chemical reaction of copper, potentially leading to learning and memory disorders by destroying synaptic plasticity [32]. Additionally, in PAH reactions, reduced BDNF expression could lead to abnormal synaptic function related to brain cognition, resulting in cognitive impairment [41]. *SCL6A3* may react with glyphosate, leading to decreased expression and increased oxidative stress in zebra fish, thus causing damage to exploration and social behavior [47]. Hence, it implies that the engagement of dyslexia-related genes in chemical reactions could contribute to dyslexia development via neuro/immune biological pathways. Further investigation is warranted to elucidate this mechanism thoroughly.

Our findings could be partially generalized to neurodevelopmental disorders, given that dyslexia risk genes are frequently expressed in the brain and contribute to brain development [58]. Based on gene expression data from the GTEx project across various tissues, dyslexia often involves susceptibility genes expressed throughout different regions of the brain [59, 60] (Table S2). Dysregulation of these genes typically results in compromised brain development [61]. For instance, alterations in the *COMT* gene have been linked to differences in brain structure evident at birth, potentially disrupting neurodevelopment and affecting cognitive abilities [62]. In addition, compounds could enter the body through various exposure routes, which also have an impact on the brain. Concurrently, we gathered 2229 ADHD susceptibility genes, 819 ID genes and 639 ASD susceptibility genes from database such as GeneCards, OMIM, NCBI, and other related sources. Upon comparing whether dyslexia risk genes were also susceptibility genes for other neurodevelopmental conditions, such as ASD, ADHD and ID, we found that the *GRIN2B* gene was a susceptibility gene for these three conditions. The *GRIN2B* gene plays a crucial role in the normal development of neurons. Mutations in human *GRIN2B* were distributed through the entire gene in a number of patients with varied neuropsychology and developmental disorders [63]. There were also 18 dyslexia susceptibility genes, two of which were concurrently susceptible. For example, *DRD3* gene may be related to the clinical significance of ASD and ADHD [64, 65]. Genes related to dyslexia and neurodevelopment usually share a common mechanism in influencing brain development, including synaptic plasticity, neuronal migration, and neurotransmitter transmission [66]. It is also common for dyslexia to co-occur with ADHD, ASD, and ID [61, 67, 68]. The joint action of relevant compounds and susceptibility genes for conditions may contribute to other neurodevelopmental disorders, but further research exploration is still needed.

A strength of our study is that, to our knowledge, it is the first to systematically investigate the overlaps between many compounds and a wide range of dyslexia risk genes. Our study may provide new links between certain chemicals and dyslexia, which could help further research on the mechanisms underlying this condition. Some limitations exist in this study. Firstly, this study only explored 95 compounds, and there may be other links between chemicals and dyslexia that have not been investigated. Secondly, the study was based on theoretical analysis and did not conduct actual clinical research, so it was uncertain if the associations between these chemicals and susceptibility genes had clinical significance. Further experimental research is needed to validate the findings.

In conclusion, our study systematically explored the overlaps between several compounds and a wide range of dyslexia susceptibility genes, providing a new perspective on the potential link between chemicals and the development of dyslexia. This study suggested that dyslexia-related genes may participate in chemical reactions of some chemicals, which may contribute to the occurrence of the condition. However, our findings are exploratory, further research is needed to validate these findings and to elucidate the mechanisms involved. Going forward, it is crucial to continue investigating the potential links between environmental factors and the development of dyslexia. This includes conducting additional animal and cellular experiments to explore the potential mechanism of dyslexia-related genes involved in chemical reactions. Additionally, further clinical research is needed to determine the clinical significance of these findings and to develop effective prevention and intervention strategies for individuals who may be at risk for dyslexia.

### Electronic supplementary material

Below is the link to the electronic supplementary material.

Supplementary Material 1

## Data Availability

Data is provided within the manuscript or supplementary information. The datasets used and/or analysed during the current study are available from the corresponding author on reasonable request.
