# Supplementary material for: Developmental dyslexia genes are selectively targeted by diverse environmental pollutants
Source: BMC Psychiatry. 2024 Jul 17;24:509. doi: 10.1186/s12888-024-05952-4 (PMC11256705; doi:10.1186/s12888-024-05952-4)
Supplement: Supplementary file 1 — Supplementary Material 1 [file 12888_2024_5952_MOESM1_ESM.docx]

**Developmental dyslexia genes are selectively targeted by diverse environmental pollutants**

**Supplementary Table 1** Compounds that have been implicated in dyslexia in epidemiological studies

**Supplementary Table 2** The basic information of 131 dyslexia risk genes

**Table S1. Compounds that have been implicated in dyslexia in epidemiological studies**

| **Source** | **Nation** | **Sample size/age (years)** | **Sample** | **Chemical** | **Classification** |
| --- | --- | --- | --- | --- | --- |
| READ (Zhu, et al.) [1] | Wuhan, Hangzhou, Jining city, China | n = 745, 7–12 | urine | thiocyanate | sodium iodide symporter (NIS) inhibitors |
| READ (Liu, et al.) [2] | Wuhan, Hangzhou, Jining city, China | n = 745, 7–12 | urine | 2-hydroxypropyl mercapturic acid (2-HPMA) | propylene oxide |
| READ (Xie, et al.) [3] | Wuhan, Hangzhou, Jining city, China | n = 845, 7–12 | urine | Diethylthiophosphate (DETP)  diethylphosphate (DEP) | organophosphate (OP) insecticides |
| Huang, et al [4] | Shantou, China | n = 116, 9.84 ± 1.23 | urine | Cobalt, zinc, and lead | metals |
| READ (Xue, et al.) [5] | China | n = 456, 9.76 ± 1.29 | urine | Selenium, argentum, | metals |
| GINIplus and LISAplus (Fuertes, et al) [6] | German | n = 4745, 10 and 15 | home address | No association between NO_2_, PM_10_, and PM_2.5_. | air pollution |
| Grant, et al. [7] | UK | n = 52, 6–14 | sweat and hair | Zinc | metals |
| Capel, et al. [8] | UK | n = 52, 11–15 | hair | Magnesium, copper, aluminum and cadmium | metals |
| Zhu, et al. [9] | Wuhan, Hangzhou, Jining city, China | n = 469, 7-12 | urine | manganese | metals |

**Supplementary table 2 The basic information of 131 dyslexia risk genes**

| **Gene symbol** | **Full name** | **Number of chemicals affecting each dyslexia gene** | **Brain expression region**^a^ | **Susceptibility genes for ASD, ADHD, or ID**^b^ |
| --- | --- | --- | --- | --- |
| *PARP1* | poly(ADP-ribose) polymerase 1 | 532 | Brain - Spinal cord (cervical c-1)  Brain - Substantia nigra | None |
| *ESR1* | estrogen receptor | 465 | Brain – Hypothalamus  Brain - Nucleus accumbens (basal ganglia) | ADHD |
| *GCLC* | glutamate-cysteine ligase catalytic subunit | 289 | Brain - Nucleus accumbens (basal ganglia)  Brain - Substantia nigra | None |
| *ESR2* | estrogen receptor 2 | 272 | Brain - Cerebellar Hemisphere  Brain – Cerebellum | ADHD |
| *SQSTM1* | sequestosome 1 | 240 | Brain - Cerebellar Hemisphere  Brain - Cerebellum | None |
| *BDNF* | brain derived neurotrophic factor | 187 | Brain - Cerebellar Hemisphere  Brain - Cerebellum | ASD ADHD |
| *CYP19A1* | cytochrome P450 family 19 subfamily A member 1 | 184 | Brain - Spinal cord (cervical c-1)  Brain – Hypothalamus | ADHD |
| *SLC2A1* | solute carrier family 2 member 1 | 153 | Brain - Spinal cord (cervical c-1)  Brain - Substantia nigra | ADHD |
| *DRD2* | dopamine receptor D2 | 126 | Brain - Nucleus accumbens (basal ganglia)  Brain - Putamen (basal ganglia) | ADHD |
| *HMGB1* | high mobility group box 1 | 119 | Brain - Cerebellar Hemisphere  Brain - Spinal cord (cervical c-1) | None |
| *MAPT* | microtubule associated protein tau | 116 | Brain – Cerebellum  Brain - Cerebellar Hemisphere | ADHD |
| *COMT* | catechol-O-methyltransferas | 112 | Brain - Spinal cord (cervical c-1)  Brain – Cerebellum | ASD ADHD |
| *IER3* | immediate early response 3 | 107 | Brain - Spinal cord (cervical c-1)  Brain – Hypothalamus | ADHD |
| *SLC6A3* | solute carrier family 6 member 3 | 100 | Brain - Substantia nigra  Brain – Hypothalamus | ID ADHD |
| *HRAS* | HRas proto-oncogene, GTPase | 98 | Brain - Cerebellar Hemisphere  Brain - Caudate (basal ganglia) | ID ADHD |
| *APOB* | apolipoprotein B | 93 | Brain - Spinal cord (cervical c-1)  Brain - Substantia nigra | None |
| *TSC22D3* | TSC22 domain family member 3 | 91 | Brain - Cerebellar Hemisphere  Brain – Cerebellum | None |
| *GRIN2B* | glutamate ionotropic receptor NMDA type subunit 2B | 86 | Brain - Frontal Cortex (BA9)  Brain - Nucleus accumbens (basal ganglia) | ASD ID ADHD |
| *SMAD2* | SMAD family member 2 | 84 | Brain - Spinal cord (cervical c-1)  Brain - Cerebellar Hemisphere | None |
| *MAP1B* | microtubule associated protein 1B | 73 | Brain - Cerebellar Hemisphere  Brain – Cerebellum | ADHD |
| *GABRA1* | gamma-aminobutyric acid type A receptor subunit alpha1 | 72 | Brain - Cerebellar Hemisphere  Brain - Frontal Cortex (BA9) | ID |
| *FLNA* | filamin A | 72 | Brain – Cerebellum  Brain - Cerebellar Hemisphere | ID |
| *SFPQ* | splicing factor proline and glutamine rich | 68 | Brain - Cerebellar Hemisphere  Brain – Cerebellum | None |
| *TAT* | tyrosine aminotransferase | 67 | Brain – Hypothalamus  Brain - Spinal cord (cervical c-1) | None |
| *SOX4* | SRY-box transcription factor 4 | 62 | Brain - Cerebellar Hemisphere  Brain – Cerebellum | None |
| *GRN* | granulin precursor | 61 | Brain - Spinal cord (cervical c-1)  Brain – Hypothalamus | None |
| *TCF4* | transcription factor 4 | 61 | Brain - Cerebellar Hemisphere  Brain – Cerebellum | ASD ID |
| *NEDD4L* | NEDD4 like E3 ubiquitin protein ligase | 60 | Brain - Cerebellar Hemisphere  Brain – Cerebellum | None |
| *GBE1* | 1,4-alpha-glucan branching enzyme 1 | 58 | Brain - Substantia nigra  Brain - Spinal cord (cervical c-1) | ID |
| *RELN* | reelin | 57 | Brain - Cerebellar Hemisphere  Brain – Cerebellum | ASD ADHD |
| *APC* | APC regulator of WNT signaling pathway | 56 | Brain - Frontal Cortex (BA9)  Brain - Anterior cingulate cortex (BA24) | None |
| *GABRG2* | gamma-aminobutyric acid type A receptor subunit gamma2 | 56 | Brain - Cerebellar Hemisphere  Brain - Frontal Cortex (BA9) | ADHD |
| *RAN* | RAN, member RAS oncogene family | 56 | Brain - Frontal Cortex (BA9)  Brain – Hypothalamus | None |
| *SEMA3C* | semaphorin 3C | 56 | Brain - Spinal cord (cervical c-1)  Brain – Hypothalamus | None |
| *DRD3* | dopamine receptor D3 | 55 | Brain - Nucleus accumbens (basal ganglia)  Brain - Caudate (basal ganglia) | ASD ADHD |
| *ELK1* | ETS transcription factor ELK1 | 51 | Brain – Cerebellum  Brain - Cerebellar Hemisphere | None |
| *VCP* | valosin containing protein | 49 | Brain - Spinal cord (cervical c-1)  Brain - Frontal Cortex (BA9) | None |
| *GABBR1* | gamma-aminobutyric acid type B receptor subunit 1 | 49 | Brain - Cerebellar Hemisphere  Brain – Cerebellum | ADHD |
| *CDK5* | cyclin dependent kinase 5 | 49 | Brain - Frontal Cortex (BA9)  Brain – Cortex | ADHD |
| *SLIT2* | slit guidance ligand | 48 | Brain - Frontal Cortex (BA9)  Brain – Cortex | None |
| *ARHGEF2* | Rho/Rac guanine nucleotide exchange factor 2 | 48 | Brain – Cerebellum  Brain - Cerebellar Hemisphere | None |
| *DLD* | dihydrolipoamide dehydrogenase | 47 | Brain - Cerebellar Hemisphere  Brain – Cerebellum | None |
| *DBH* | dopamine beta-hydroxylase | 47 | Brain – Cortex  Brain - Anterior cingulate cortex (BA24) | ASD ADHD |
| *CACNA1H* | calcium voltage-gated channel subunit alpha1 H | 46 | Brain - Nucleus accumbens (basal ganglia)  Brain - Caudate (basal ganglia) | ASD ADHD |
| *FOXP2* | forkhead box P2 | 45 | Brain - Frontal Cortex (BA9)  Brain – Hypothalamus | ASD ADHD |
| *PSEN1* | presenilin 1 | 44 | Brain - Spinal cord (cervical c-1)  Brain - Substantia nigra | ADHD |
| *NSF* | N-ethylmaleimide sensitive factor, vesicle fusing ATPase | 44 | Brain - Frontal Cortex (BA9)  Brain - Cerebellar Hemisphere | None |
| *PDE7B* | phosphodiesterase 7B | 44 | Brain - Nucleus accumbens (basal ganglia)  Brain - Caudate (basal ganglia) | None |
| *PLA2G6* | phospholipase A2 group VI | 43 | Brain - Spinal cord (cervical c-1)  Brain – Cortex | ID |
| *GABRB3* | gamma-aminobutyric acid type A receptor subunit beta3 | 43 | Brain - Frontal Cortex (BA9)  Brain - Cerebellar Hemisphere | ASD ADHD |
| *PCSK6* | proprotein convertase subtilisin/kexin type 6 | 42 | Brain - Spinal cord (cervical c-1)  Brain - Substantia nigra | None |
| *ROBO1* | roundabout guidance receptor 1 | 42 | Brain - Nucleus accumbens (basal ganglia)  Brain - Cerebellar Hemisphere | ADHD |
| *TLE3* | TLE family member 3, transcriptional corepressor | 40 | Brain – Cerebellum  Brain - Cerebellar Hemisphere | None |
| *FRAXA* | fragile site, folic acid type, rare, fra (X) (q27.3) A | 40 | Brain - Spinal cord (cervical c-1)  Brain - Cerebellar Hemisphere | ADHD |
| *VAPA* | VAMP associated protein A | 39 | Brain - Cerebellar Hemisphere  Brain - Spinal cord (cervical c-1) | None |
| *DRD4* | dopamine receptor D4 | 39 | Brain – Cerebellum  Brain - Cerebellar Hemisphere | ASD ADHD |
| *TCF12* | transcription factor 12 | 38 | Brain - Spinal cord (cervical c-1)  Brain – Cerebellum | ID |
| *SARDH* | sarcosine dehydrogenase | 37 | Brain – Cortex  Brain - Anterior cingulate cortex (BA24) | None |
| *TREM2* | triggering receptor expressed on myeloid cells 2 | 36 | Brain - Spinal cord (cervical c-1)  Brain - Substantia nigra | None |
| *NRGN* | neurogranin | 36 | Brain - Frontal Cortex (BA9)  Brain – Cortex | ASD ADHD |
| *STIM1* | stromal interaction molecule 1 | 35 | Brain – Cerebellum  Brain - Cerebellar Hemisphere | None |
| *BCL11A* | BCL11 transcription factor A | 35 | Brain - Frontal Cortex (BA9)  Brain – Cortex | ASD ADHD |
| *MYO5B* | myosin V | 34 | Brain - Frontal Cortex (BA9)  Brain - Anterior cingulate cortex (BA24) | None |
| *TMEM106B* | transmembrane protein 106B | 33 | Brain - Cerebellar Hemisphere  Brain - Spinal cord (cervical c-1) | None |
| *SRF* | serum response factor | 33 | Brain – Cerebellum  Brain - Cerebellar Hemisphere | None |
| *SHC2* | SHC adaptor protein 2 | 32 | Brain – Cortex  Brain – Hypothalamus | None |
| *DOCK4* | dedicator of cytokinesis 4 | 32 | Brain – Cerebellum  Brain - Nucleus accumbens (basal ganglia) | ADHD |
| *EVA1A* | eva-1 homolog A, regulator of programmed cell death | 32 | Brain - Caudate (basal ganglia)  Brain - Putamen (basal ganglia) | None |
| *AP2M1* | adaptor related protein complex 2 subunit mu 1 | 32 | Brain - Frontal Cortex (BA9)  Brain – Cortex | ADHD |
| *GTF2I* | general transcription factor II | 31 | Brain - Cerebellar Hemisphere  Brain – Cerebellum | None |
| *STUB1* | STIP1 homology and U-box containing protein 1 | 31 | Brain - Cerebellar Hemisphere  Brain - Frontal Cortex (BA9) | ID |
| *RBFOX2* | INTS3 and NABP interacting protein | 30 | Brain - Cerebellar Hemisphere  Brain – Cerebellum | None |
| *CNTNAP2* | contactin associated protein | 30 | Brain - Frontal Cortex (BA9)  Brain - Spinal cord (cervical c-1) | ASD ADHD |
| *MRPL19* | mitochondrial ribosomal protein L19 | 30 | Brain - Spinal cord (cervical c-1)  Brain - Frontal Cortex (BA9) | ADHD |
| *CMIP* | c-Maf inducing protein | 30 | Brain - Frontal Cortex (BA9)  Brain – Cortex | ADHD |
| *GPSM2* | G protein signaling modulator 2 | 30 | Brain - Spinal cord (cervical c-1)  Brain - Substantia nigra | ID |
| *RTN4R* | reticulon 4 receptor | 30 | Brain – Cerebellum  Brain - Cerebellar Hemisphere | ADHD |
| *ACOT13* | acyl-CoA thioesterase 13 | 29 | Brain - Frontal Cortex (BA9)  Brain - Cerebellar Hemisphere | ADHD |
| *NDEL1* | nudE neurodevelopment protein 1 like 1 | 28 | Brain - Cerebellar Hemisphere  Brain - Frontal Cortex (BA9) | None |
| *ORAI1* | ORAI calcium release-activated calcium modulator 1 | 27 | Brain - Spinal cord (cervical c-1)  Brain - Substantia nigra | None |
| *DCDC2* | doublecortin domain containing 2 | 26 | Brain – Hypothalamus  Brain - Frontal Cortex (BA9) | ADHD |
| *DGKI* | diacylglycerol kinase iota | 26 | Brain - Cerebellar Hemisphere  Brain – Cerebellum | None |
| *OTX1* | orthodenticle homeobox 1 | 26 | Brain – Cortex  Brain - Anterior cingulate cortex (BA24) | ASD |
| *PCNT* | pericentrin | 26 | Brain – Cerebellum  Brain - Cerebellar Hemisphere | ID ADHD |
| *CCDC136* | coiled-coil domain containing 136 | 25 | Brain - Cerebellar Hemisphere  Brain – Cerebellum | None |
| *PNKP* | polynucleotide kinase 3'-phosphatase | 25 | Brain – Cerebellum  Brain - Cerebellar Hemisphere | ID |
| *ELK3* | ETS transcription factor ELK | 25 | Brain - Cerebellar Hemisphere  Brain – Cerebellum | None |
| *GNPTAB* | N-acetylglucosamine-1-phosphate transferase subunits alpha and beta | 24 | Brain - Frontal Cortex (BA9)  Brain - Cerebellar Hemisphere | ID |
| *SEMA4F* | ssemaphorin 4F | 24 | Brain – Cerebellum  Brain - Cerebellar Hemisphere | None |
| *DNAH5* | dynein axonemal heavy chain 5 | 23 | Brain – Hypothalamus  Brain - Nucleus accumbens (basal ganglia) | ASD |
| *MOG* | myelin oligodendrocyte glycoprotein | 22 | Brain - Spinal cord (cervical c-1)  Brain - Substantia nigra | ASD ADHD |
| *CEP63* | centrosomal protein 63 | 22 | Brain - Cerebellar Hemisphere  Brain – Cerebellum | None |
| *NAGPA* | N-acetylglucosamine-1-phosphodiester alpha-N-acetylglucosaminidase | 21 | Brain – Cortex  Brain - Frontal Cortex (BA9) | None |
| *CHMP2B* | charged multivesicular body protein 2B | 20 | Brain - Spinal cord (cervical c-1)  Brain - Substantia nigra | None |
| *EPM2A* | EPM2A glucan phosphatase, laforin | 20 | Brain - Spinal cord (cervical c-1)  Brain - Substantia nigra | None |
| *ATP2C2* | ATPase secretory pathway Ca2+ transporting 2 | 19 | Brain – Hypothalamus  Brain – Amygdala | ADHD |
| *EBPL* | EBP like | 19 | Brain – Hypothalamus  Brain - Cerebellar Hemisphere | None |
| *JRK* | Jrk helix-turn-helix protein | 18 | Brain – Cerebellum  Brain - Cerebellar Hemisphere | ADHD |
| *TDP2* | tyrosyl-DNA phosphodiesterase | 18 | Brain - Cerebellar Hemisphere  Brain – Cerebellum | ADHD |
| *GCFC2* | GC-rich sequence DNA-binding factor 2 | 18 | Brain - Cerebellar Hemisphere  Brain – Cerebellum | ADHD |
| *C9orf72* | C9orf72-SMCR8 complex subunit | 17 | Brain - Cerebellar Hemisphere  Brain – Cerebellum | ADHD |
| *GNPTG* | N-acetylglucosamine-1-phosphate transferase subunit gamma | 17 | Brain - Spinal cord (cervical c-1)  Brain - Cerebellar Hemisphere | ID |
| *KCNH8* | potassium voltage-gated channel subfamily H member 8 | 17 | Brain - Spinal cord (cervical c-1)  Brain – Hippocampus | None |
| *NRSN1* | neurensin 1 | 17 | Brain - Frontal Cortex (BA9)  Brain - Anterior cingulate cortex (BA24) | None |
| *PCDH11X* | protocadherin 11 X-linked | 17 | Brain - Nucleus accumbens (basal ganglia)  Brain – Hypothalamus | None |
| *NIPA2* | NIPA magnesium transporter 2 | 17 | Brain - Cerebellar Hemisphere  Brain - Frontal Cortex (BA9) | ADHD |
| *DIP2A* | disco interacting protein 2 homolog A | 17 | Brain – Cerebellum  Brain - Cerebellar Hemisphere | ADHD |
| *INIP* | RNA binding fox-1 homolog 2 | 16 | Brain - Cerebellar Hemisphere  Brain - Frontal Cortex (BA9) | None |
| *DNAH11* | dynein axonemal heavy chain 11 | 16 | Brain – Hippocampus  Brain - Caudate (basal ganglia) | None |
| *ARSD* | arylsulfatase D | 16 | Brain – Hypothalamus  Brain – Cerebellum | None |
| *DYM* | dymeclin | 15 | Brain - Cerebellar Hemisphere  Brain – Cerebellum | None |
| *SLC2A4RG* | SLC2A4 regulator | 15 | Brain – Cerebellum  Brain - Cerebellar Hemisphere | None |
| *HTR1F* | 5-hydroxytryptamine receptor 1F | 15 | Brain - Frontal Cortex (BA9)  Brain – Cortex | None |
| *RFX1* | regulatory factor X | 14 | Brain – Cerebellum  Brain - Cerebellar Hemisphere | None |
| *KCNH4* | potassium voltage-gated channel subfamily H member 4 | 13 | Brain - Caudate (basal ganglia)  Brain - Putamen (basal ganglia) | None |
| *ZNF280D* | zinc finger protein 280D | 13 | Brain - Cerebellar Hemisphere  Brain – Cerebellum | None |
| *DNAAF4* | dynein axonemal assembly factor 4 | 12 | Brain – Hippocampus  Brain - Caudate (basal ganglia) | ADHD |
| *MYO18B* | myosin XVIIIB | 12 | Brain – Hypothalamus  Brain – Cortex | None |
| *ZNF804A* | zinc finger protein 804 | 12 | Brain - Cerebellar Hemisphere  Brain – Cerebellum | ASD ADHD |
| *CNTNAP5* | contactin associated protein family member 5 | 12 | Brain - Frontal Cortex (BA9)  Brain – Cortex | ADHD |
| *MC5R* | melanocortin 5 receptor | 12 | Brain - Anterior cingulate cortex (BA24)  Brain - Frontal Cortex (BA9) | None |
| *KIAA0319* | KIAA0319 | 11 | Brain - Cerebellar Hemisphere  Brain – Cerebellum | ADHD |
| *KIAA0319L* | KIAA0319 like | 11 | Brain - Cerebellar Hemisphere  Brain – Cerebellum | ADHD |
| *OPA3* | outer mitochondrial membrane lipid metabolism regulator OPA3 | 10 | Brain - Cerebellar Hemisphere  Brain – Cerebellum | ADHD |
| *RHD* | Rh blood group D antigen | 10 | Brain – Cerebellum  Brain - Cerebellar Hemisphere | None |
| *STATH* | statherin | 9 | —— | None |
| *ZNF385D* | zinc finger protein 385D | 8 | Brain - Cerebellar Hemisphere  Brain – Cerebellum | ADHD |
| *RHCE* | Rh blood group CcEe antigen | 4 | Brain - Spinal cord (cervical c-1)  Brain - Nucleus accumbens (basal ganglia) | None |
| *TRD* | T cell receptor delta locus | 1 | Brain – Cerebellum  Brain - Cerebellar Hemisphere | None |
| [*PCDHG*](mailto:PCDHG@) | protocadherin gamma cluster | 1 | Brain – Cerebellum  Brain - Cerebellar Hemisphere | None |

Abbreviation: ASD: Autism Spectrum Disorder; ADHD: Attention-Deficit/Hyperactivity Disorder; ID: Intellectual Disability

^a^ The data on gene expression regions in the brain was obtained from Genotype-Tissue Expression project.

^b^ The association between these genes and ASD, ADHD, and ID was obtained from GeneCards, OMIM, NCBI, and other related source.

Reference

1. Zhu K, Wan Y, Zhu B, Wang H, Liu Q, Xie X, Jiang Q, Feng Y, Xiao P, Xiang Z *et al*: **Association of perchlorate, thiocyanate, and nitrate with dyslexic risk**. *Chemosphere* 2023, **325**:138349.

2. Liu Q, Wan Y, Zhu B, Xie X, Zhu K, Jiang Q, Feng Y, Xiao P, Xiang Z, Wu X *et al*: **Association between urinary propylene oxide metabolite and the risk of dyslexia**. *Environ Pollut* 2022, **292**(Pt B):118469.

3. Xie X, Wan Y, Zhu B, Liu Q, Zhu K, Jiang Q, Feng Y, Xiao P, Wu X, Zhang J *et al*: **Association between urinary dialkylphosphate metabolites and dyslexia among children from three cities of China: The READ program**. *Sci Total Environ* 2022, **814**:151852.

4. Huang A, Zhang J, Wu K, Liu C, Huang Q, Zhang X, Lin X, Huang Y: **Exposure to multiple metals and the risk of dyslexia - A case control study in Shantou, China**. *Environ Pollut* 2022, **307**:119518.

5. Xue Q, Zhou Y, Gu H, Xie X, Hou F, Liu Q, Wu H, Zhu K, Wan Z, Song R: **Urine metals concentrations and dyslexia among children in China**. *Environ Int* 2020, **139**:105707.

6. Fuertes E, Standl M, Forns J, Berdel D, Garcia-Aymerich J, Markevych I, Schulte-Koerne G, Sugiri D, Schikowski T, Tiesler CM *et al*: **Traffic-related air pollution and hyperactivity/inattention, dyslexia and dyscalculia in adolescents of the German GINIplus and LISAplus birth cohorts**. *Environ Int* 2016, **97**:85-92.

7. Grant EC, Howard JM, Davies S, Chasty H, Hornsby B, Galbraith J: **Zinc deficiency in children with dyslexia: concentrations of zinc and other minerals in sweat and hair**. *Br Med J (Clin Res Ed)* 1988, **296**(6622):607-609.

8. Capel ID, Pinnock MH, Dorrell HM, Williams DC, Grant EC: **Comparison of concentrations of some trace, bulk, and toxic metals in the hair of normal and dyslexic children**. *Clin Chem* 1981, **27**(6):879-881.

9. Zhu K, Liu Q, Xie X, Jiang Q, Feng Y, Xiao P, Wu X, Zhu B, Song R: **Interaction between manganese and SLC6A3 genetic polymorphisms in relation to dyslexia**. *Neurotoxicology* 2022, **92**:102-109.
